# tissueloc: Whole slide digital pathology image tissue localization

**DOI:** 10.21105/joss.01148

**Published:** 2019-01-02

**Authors:** Pingjun Chen, Lin Yang

**Affiliations:** 1 J. Crayton Pruitt Family Department of Biomedical Engineering, University of Florida

## Background

tissueloc is an open-source Python package for fast and accurate tissue localization on whole slide image (WSI). Automatic pathology diagnosis using WSI gradually becomes a research hotspot in biomedical imaging domain (Cruz-Roa et al., 2014, Barker, Hoogi, Depeursinge, & Rubin (2016)). Because of the gigabyte size of WSI, instead of directly taking the WSI as input, patch-based strategy is commonly used to deal with WSI (Cruz-Roa et al., 2014, Hou et al. (2016)). As there are large amounts of background regions that are useless for diagnosis, researchers working on automatic WSI diagnosis can utilize tissueloc to locate genuine tissue regions and focus their analysis on these regions.

## Overview

tissueloc mainly contains two functionalities: selecting proper low level image from WSI and tissue localization based on the selected low level WSI.

The width and height of WSI are far larger than 10,000 pixels. Locating tissue regions directly on WSI image is computationally expensive. However, based on the pyramid storage structure of WSI, we can select a proper low level image from the WSI for following tissue localization. The low level slide image can have much smaller size, thus can speed up the tissue localization process. Based on the setting of maximum width or height of the low level image, we select the level that its corresponding image has size smaller but closest to the setting.

Tissue localization is applied on the selected low level image based on a series of basic image processing techniques. The main procedures include: 1) Low level WSI loading. 2) Color space conversion from RGB to gray. 3) Inverse binarization to generate binary image. 4) Hole filling of the binary image. 5) Small object removal. 6) Contour finding.

The proposed WSI tissue localization is very efficient as it is entirely based on basic image processing techniques and applied on low level image, which could act as a preprocessing step for automatic WSI analysis. Researchers can focus their analysis on those patches inside the located tissue regions and avoid those irrelevant background regions.

## Figures and Tables

**Figure 1:**
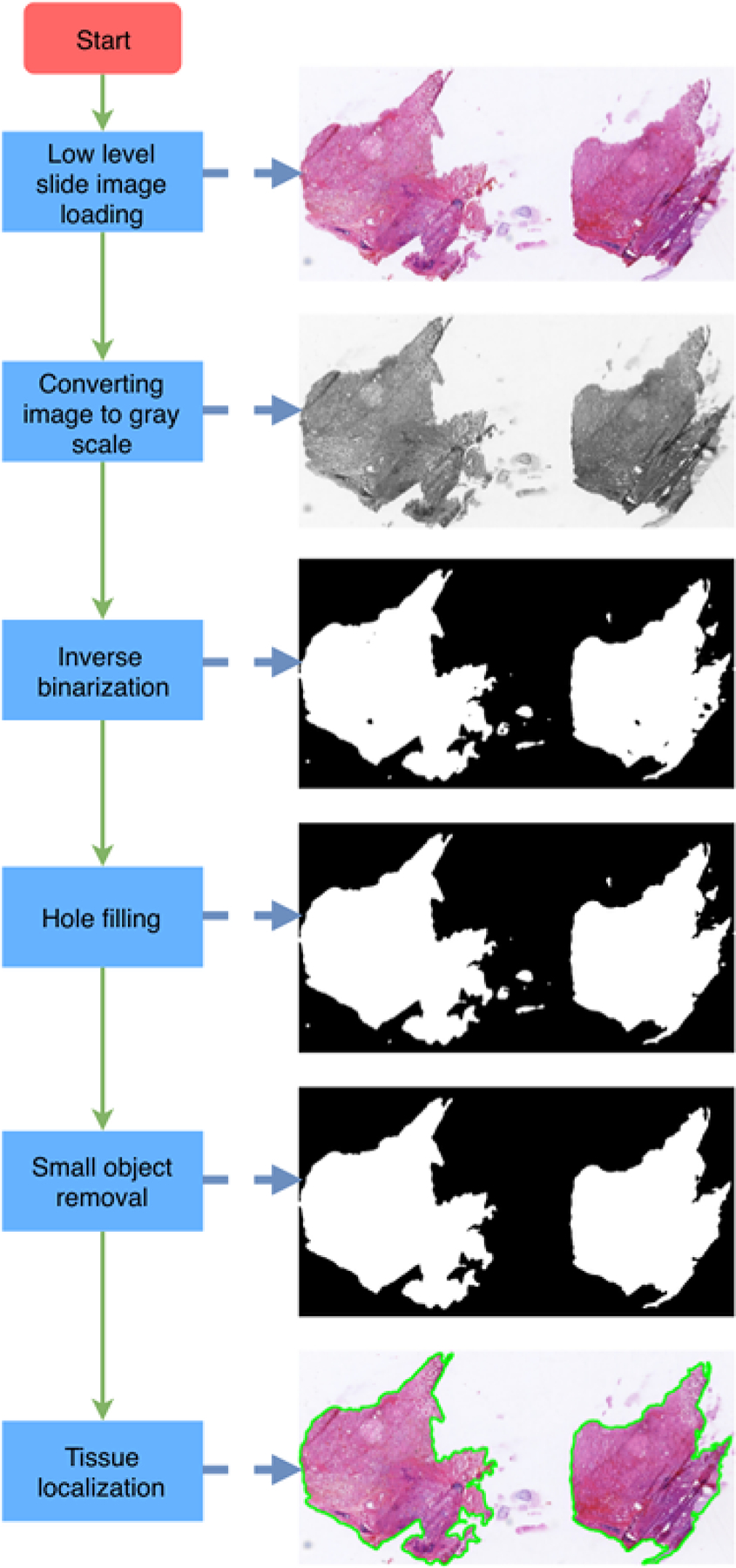
Tissue localization pipeline for whole slide image. The main procedures include: 1) Low level image loading. 2) Color space conversion. 3) Inverse binarization. 4) Hole filling. 5) Small object removal. 6) Contour finding.
